# Supplementary material for: Efficient neural spike sorting using data subdivision and unification
Source: PLoS One. 2021 Feb 10;16(2):e0245589. doi: 10.1371/journal.pone.0245589 (PMC7875432; doi:10.1371/journal.pone.0245589)
Supplement: Attachment — Submitted filename: Response to reviewers.pdf [file pone.0245589.s002.pdf]

PONE-D-19-23638

Efficient Neural Spike Sorting using Data Subdivision and Unification

PLOS ONE

To the Editor,

Gennady Cymbalyuk
Academic Editor, PLOS ONE

We would like to acknowledge and appreciate the efforts and time of the editor and the reviewers for their invaluable comments and suggestions that has allowed us to enhance the quality of our manuscript.

Below are the suggested revisions according to valuable comments from the reviewers.

1) Abstract.

1. **What do you mean by "big data dynamics"? This term is ambiguous. Please change this sentence and the next one, immediately following. Rephrase in order to avoid reference to other algorithms reported in the literature if the Authors do not cite explicitly which ones. The current reference is too general and inappropriate.**

   Author Response: The manuscript has been updated according to reviewers comment (Line 16 to 20).

2. **How many datasets did you use?**

   Author Response: The manuscript has been updated according to reviewers comment (Line 24 to 26).

2) Introduction.

Please, focus the introduction on the problems addressed and thoroughly review the literature and the current state of the art in the field.

1. **The review of the literature is not complete, because it missed one key important paper related to this topic, in particular because that paper has introduced for the first time a series of steps that are very close, if not identical, to the steps of data subdivision, clusters formed for each sub-set, unification process by merging neighbor clusters in feature space, thus achieving unified clusters in the end. This paper is the following:**

   Aksenova TI, Chibirova OK, Dryga OA, Tetko IV, Benabid AL, Villa AE. An unsupervised automatic method for sorting neuronal spike waveforms in awake and freely moving animals. Methods. 2003; 30(2):178-187. doi: 10.1016/S1046-2023(03)00079-3 : this is the

very first paper (2003) to describe unsupervised neural spike sorting based on a fast implementation suitable for real-time application for high-density neural probes.

Author Response: The manuscript has been updated according to reviewers comment (Line 232 to 234).

2. **With respect to application of spike sorting to online experimental procedures, the Authors should also mention:**

   a) **Abeles M, Goldstein MH. Multispike train analysis. Proceedings of the IEEE. 1977; 65(5):762-773. doi:10.1109/PROC.1977.10559 : this is a seminal paper (1977) for detecting and identifying the spikes in multispike trains based on signal detection by template matching.**

      Author Response: The manuscript has been updated according to reviewers comment (Line 87 to 90).

   b) **Wouters J, Kloosterman F, Bertrand A. Towards online spike sorting for high-density neural probes using discriminative template matching with suppression of interfering spikes. J Neural Eng. 2018; 15(5):056005. doi: 10.1088/1741-2552/aace8a : a fast and computationally cheap method for real-time applications**.

      Author Response: The manuscript has been updated according to reviewers comment (Line 109 to 111).

3. **Consider recently developed spike sorting algorithms :**

   Chung, Jason E., Jeremy F. Magland, Alex H. Barnett, Vanessa M. Tolosa, Angela C. Tooker, Kye Y. Lee, Kedar G. Shah, Sarah H. Felix, Loren M. Frank, and Leslie F. Greengard. "A fully automated approach to spike sorting." Neuron 95, no. 6 (2017): 1381-1394.

   Author Response: The manuscript has been updated according to reviewers comment (Line 123 to 127).

4. **A more satisfactory review of the literature should also include:**

   Zamani M, Demosthenous A. (2014) Feature extraction using extrema sampling of discrete derivatives for spike sorting in implantable upper-limb neural prostheses. IEEE Trans Neural Syst Rehabil Eng. 2014 Jul;22(4):716-726. doi: 10.1109/TNSRE.2014.2309678.

   Author Response: The manuscript has been updated according to reviewers comment (Line 95 to 98).

3) Materials and Methods.

The Authors mention several times the problem of noisy recordings, but they do not examine which types of noise --and/or artifacts-- are present and the methods to face this problem that have been described in the recent literature. A better way to compare the methods presented by the Authors in their Table 2 and Table 3 could have been to add several known levels of noise to the same benchmarked data set and see how performances and accuracies allow to discriminate the most robust algorithms.

1. **To this end, the Authors should consider these papers:**

   Choi JH, Jung HK, Kim T. (2006) A new action potential detector using the MTEO and its

effects on spike sorting systems at low signal-to-noise ratios. IEEE Trans Biomed Eng. 2006 Apr;53(4):738-46. doi: 10.1109/TBME.2006.870239

Paralikar KJ, Rao CR, Clement RS. (2009) New approaches to eliminating common-noise artifacts in recordings from intracortical microelectrode arrays: inter-electrode correlation and virtual referencing. J Neurosci Methods. 2009 Jun 30;181(1):27-35. doi: 10.1016/j.jneumeth.2009.04.014.

Pillow JW1, Shlens J, Chichilnisky EJ, Simoncelli EP. (2013) A model-based spike sorting algorithm for removing correlation artifacts in multi-neuron recordings. PLoS One. 2013 May 3;8(5):e62123. doi: 10.1371/journal.pone.0062123.

Takekawa T, Ota K, Murayama M, Fukai T. (2014) Spike detection from noisy neural data in linear-probe recordings. Eur J Neurosci. 2014 Jun;39(11):1943-50. doi: 10.1111/ejn.12614: an older reference to Takekawa is provided but it should be replaced by this one .

Author Response: The manuscript has been updated according to reviewers comment (Line 74 to 87).

2. **The Authors discuss Spike sorting accuracy (Subsection 3.5) but false alarm ratio is also an extremely important feature to be considered (and discussed in several papers cited above) for the evaluation of the quality of neural spike sorting.**

   Author Response: The manuscript has been updated according to reviewers comment (Line 270 to 286).

4) Results.

1. **The Authors should provide the MATLAB codes, with the description of the MATLAB version and environment, of their algorithms. They compare many methods developed elsewhere and it is of paramount importance to assess that the Authors' implementation follows exactly the algorithms cited in the literature.**

   Author Response: The manuscript has been updated according to reviewers comment (Line 316 to 324).

2. **A test against a surrogate data set could also be informative for the readers to be convinced of their superior efficiency in the spike sorting procedure claimed by the Authors.**

   Author Response: The manuscript has been updated according to reviewers comment (Line 250 to 256).

3. **Optimal length: describe how relevant it is to have the 'optimal length'. What I missed here is a discussion of how relevant it is to have the 'optimal length'. Can I be off by a factor of 2 and it doesn't really matter?**

   Author Response: The manuscript has been updated according to reviewers comment (Line 205 to 209).

4. **Please, substantiate 'OL parameter is dependent on the algorithm type rather than on the data dynamics.' The spiking rates may vary by 2 orders of magnitude, so you may end up with clusters that simply don't have enough spikes?**

   Author Response: The manuscript has been updated according to reviewers comment (Line 228 to 231).

5. **Clarify labeling in Figure 4., Labelling in Figure 4 is messy, I don't understand what is plotted.**

   Author Response: The figure has been updated according to reviewers comment (Figure 4).

5) **Unification of subclusters:**
   Describe in detail how you account for differing variances in different dimensions (i.e. principal components). Explain what 'the standard distribution and normal distribution curves' are. In general, describe how this technique is applied to the data. Do you apply it to sequential segments, blocks of segments or pairwise across the recording?

   I don't understand how you account for differing variances in different dimensions (i.e. principal components). And for distances, in 1D, the 95% claim is valid, but here you're talking about volumes. And I'm completely lost about what 'the standard distribution and normal distribution curves' are.
   In general, I'm wondering how this technique is applied to the data. Do you apply it to sequential segments, blocks of segments or pairwise across the recording?

   Author Response: The manuscript has been updated according to reviewers comment (Line 220 to 228).

6) **Performance evaluation: Why do you choose two examples where both the conventional and your method do not work for showing performance improvement?**

   Author Response: The manuscript has been updated according to reviewers comment (Line 288 to 295).

7) **Figure 6: Why those spline fits? Suggests that the different methods are related, please, explain.**

   Author Response: The figure has been updated according to reviewers comment (Figure 6).

8) **Table 3: Numbers suggest a very high accuracy, and no error estimate is given. How did you achieve such a high precision? K-means for example is known to give very different results in different runs. Are these averages over multiple runs? And does the K-means example involve multiple runs to obtain stable clusters? Which of these algorithms converge to the same result every time they are run? Could part of your accuracy improvement be due to running K-means more often, effectively averaging results?**

   Author Response: The manuscript has been updated according to reviewers comment (Line 309 to 313).

9) **Figure 7: Lines/symbols are overlapping to an extent that this figure becomes uninformative. Maybe separate plots or cluster centroids for different segments? Please, provide a plot showing the temporal stationarity of firing rates (for different segments).**

   Author Response: The figures has been updated according to reviewer's comment (Figure 7 and Figure 8).

10) **Temporal speedup: Please, clarify description of the algorithm concerning temporal speedup. What is the advantage of independent clustering? How does your method compare to a density based approach?**

If I understood things correctly (and I'm not sure I did), PCA/Wavelet is run on the whole dataset to obtain low dimensional representations of spikes. Then batches of N spikes are clustered. That sounds similar to what Kilosort does, except that batches are used for optimizing clusters rather than clustering them independently. What is the advantage of independent clustering? Mountainsort on the other hand follows a density based approach, which also seems to scale quite well with recording size. How does your method compare to a density based approach?

Author Response: The manuscript has been updated according to reviewers comment (Line 149 to 157).

11) **Clustering accuracy: The measure you are using puts a higher weight on large clusters with a lot of spikes. In many datasets, these are multiunit clusters that are hard to separate. It would be nice to have some measure of temporal stationarity.**

Author Response: The manuscript has been updated according to reviewers comment (Line 221 to 231).

Journal Requirements:

When submitting your revision, we need you to address these additional requirements.

1) Please ensure that your manuscript meets PLOS ONE's style requirements, including those for file naming. The PLOS ONE style templates can be found at

Author Response: The manuscript is according to the style requirements of PLOS One Journal.

2) Thank you for stating the following in the Acknowledgments Section of your manuscript:

[The research work is fully supported by Neural and Cognitive Systems Lab at Institute for Intelligent Systems Research and Innovation, Deakin University.]

We note that you have provided funding information that is not currently declared in your Funding Statement. However, funding information should not appear in the Acknowledgments section or other areas of your manuscript. We will only publish funding information present in the Funding Statement section of the online submission form.

Please remove any funding-related text from the manuscript and let us know how you would like to update your Funding Statement. Currently, your Funding Statement reads as follows:

[The author(s) received no specific funding for this work.]

Please include the updated Funding Statement in your cover letter. We will change the online submission form on your behalf.

Author Response: Funding related text is removed from the manuscript. We don't require any updates in the funding statement.

Thanks

Asim Bhatti
