# Supplementary material for: Efficient neural spike sorting using data subdivision and unification
Source: PLoS One. 2021 Feb 10;16(2):e0245589. doi: 10.1371/journal.pone.0245589 (PMC7875432; doi:10.1371/journal.pone.0245589)
Supplement: Attachment — Submitted filename: Rebuttal Letter_R2.pdf [file pone.0245589.s003.pdf]

PONE-D-19-23638

Efficient Neural Spike Sorting using Data Subdivision and Unification

To the Editor,

Prof Gennady Cymbalyuk
Academic Editor, PLOS ONE

We would like to acknowledge and appreciate the efforts and time of the editor and the reviewers for their invaluable comments and suggestions that has allowed us to enhance the quality of our manuscript.

Below are the revisions according to valuable comments from the reviewers.

- **Figure 7: Lines/symbols are overlapping to an extent that this figure becomes uninformative. Maybe separate plots or cluster centroids for different segments? Please, provide a plot showing the temporal stationarity of firing rates (for different segments).**

  Author Response: New figures (Fig 8 and Fig 09) are introduced clearly highlighting the performance difference between conventional and proposed algorithm in terms of computational efficiency and clustering accuracy. Separate cluster of different data segments are shown. Clustering outcome of intermediate steps is also shown to facilitate readers' understanding of the proposed algorithm.

- **Temporal speedup: Please, clarify description of the algorithm concerning temporal speedup. What is the advantage of independent clustering? How does your method compare to a density based approach?**

  Author Response: A new figure (Figure 4) is added to address this comment, highlighting the differentiation between conventional spike sorting and proposed spike sorting mechanisms. Please refer to lines 132-140, and Table 1 for explanation on the effect of data size on the computational efficiency and temporal speedup of the clustering algorithms. In addition, please refer to lines 168-186 highlighting the comparison between density based approach and proposed method.

- **Clustering accuracy: The measure you are using puts a higher weight on large clusters with a lot of spikes. In many datasets, these are multiunit clusters that are hard to separate. It**

would be nice to have some measure of temporal stationarity.

Author Response: Please refer to accuracy index "A" highlighted in expression (4) and explanation in lines 290-307 where estimation of clustering accuracy is defined. Accuracy index is defined as the percentage of spikes accurately assigned to the relevant cluster, as per the ground truth, with respect to total number of spikes. This makes the accuracy index "A" independent of the number of spikes or the size of the cluster. As the contributing of the proposed mechanism is data processing rather than the clustering, which is adopted from conventional algorithms, therefore commenting on the temporal stationarity is out of the scope of this work.

- **'The surrounding region between −2SD to 2SD, containing about 95 percent of the cluster data...' This statement is still wrong.**

  What are 'Quirogo datasets'?

  Author Response: The manuscript has been updated according to reviewers comment (Line 24 to 26). The reference of the adopted data set and brief explanation is also provide in lines 267-272.

  In addition the statement in reference to 2SD is updated, lines 246-247.

Thanks

Asim Bhatti
