# Supplementary material for: Efficient neural spike sorting using data subdivision and unification
Source: PLoS One. 2021 Feb 10;16(2):e0245589. doi: 10.1371/journal.pone.0245589 (PMC7875432; doi:10.1371/journal.pone.0245589)
Supplement: Attachment — Submitted filename: PONE-D-19-23638-R3-Response to Reviewers.pdf [file pone.0245589.s004.pdf]

PONE-D-19-23638-R3

Efficient Neural Spike Sorting using Data Subdivision and Unification

PLOS ONE

To the Editor,

Prof Alexandros Iosifidis
Academic Editor, PLOS ONE

We would like to acknowledge and appreciate the efforts and time of the editor and the reviewers for their invaluable comments and suggestions that has allowed us to enhance the quality of our manuscript.

Below are the suggested revisions according to valuable comments from the reviewers.

1) I think I'm still missing some crucial information about the analysis. First I thought, that the performance improvement was somewhat related to no stationarities in the data and you have shown (great, thanks) that this is clearly not the case. Another thing that I kept pointing out in my reviews and is still somewhat misleading in the presentation of the method is that in a high dimensional multivariate gaussian distribution, the probability for a datapoint to be within a 2 sigma radius from the center is not 95% but rather dependent on the number of dimensions, i.e. at most (95%)^d (for L1 norm), where d is the number of dimensions (or PCA components/ features).

   Author Response: The manuscript is updated with additional information, mathematical expressions and references to address the points raised by the review as well as for the ease of the general readers (Line 237 to 252).

2) I haven't really found the number of dimensions you used in the paper (and you really do need to report it, it is a crucial number), but there is one figure suggesting the use of 10 features/dimensions. This seems high to me (and you may want to discuss such a parameter choice in the Discussion), what would have expected from other work would be 3-4 features.

   In any case, in the 10 feature case, your 2 sigma radius then accounts for at most 60% of the datapoints, so there are a lot of points outside your cluster boundaries. Does that explain why

those widely used algorithms are working so poorly? If so, that's fine, but you want to discuss it in the Discussion section. It is also not clear to me how different dimensions are handled and you should elaborate a bit on that in the Methods. Is each dimension scaled such that variances match? If that is the case you're down weighting the first principal component and effectively explaining noisy, low variance features? Or am I missing something more subtle? You're reporting a performance improvement and I still don't see any reason why this should happen and especially why it would happen so consistently, given that all these algorithms have been used very successfully for years. I'm totally fine with the speed improvement and follow the argument that this should happen. But a general classification performance improvement is very hard to believe, so you need to at least report the specific circumstances under which it happens, i.e. the number of features/ dimensions and make clear that you're potentially inflating tiny differences in principal components with small variances (unless you corrected for that in some way, in that case it should be reported). Ideally, you should have some idea about a mechanism for the performance improvement and discuss it in the Discussion (is it some kind of regularization effect that would be beneficial for noisy data?). Specifically, do report the number of features/PCA components used.

Do make clear whether the standard deviation was estimated for each component separately, thus enhancing the effect of small components, or whether (and how) you accounted for differences in the variances of features/PCA components. Ideally, specify a typical variation between variances of the features/PCA components (e.g. ratio between largest and smallest) and mention whether the results were sensitive to the number of PCA components. A thorough analysis of the effect of dimensionality and scaling is certainly beyond the scope of this article, but I'm sure you made observations what happens if you change these parameters. You shall discuss them in the Discussion, and maybe even speculate about a mechanism or a scenario that tends to give performance improvements.

Author Response: Author Response: Further explanation is added, please refer to lines 282 to 298.

3) Figure 7 has errorbars now, so please mention briefly how you obtained them/what they reflect. Further, numbers reported suggest a huge precision in comparison to these errorbars. Please round them, and wherever refered to in the text, add the uncertainty in brackets (e.g. 53+-6 %). You may leave the uncertainty in the table for clarity as it is already shown in Figure 7.

   Author Response: Taking into account reviewer's comment, Figure 7 is updated to provide simplified performance comparison. Performance outcomes, averaged over 10 repetitions, are presented for simplicity and ease of understanding.

4) **Other remarks**

   Figure 8+9: markers and labels don't match.
   ln65: Brain consists
   ln105: automatically estimate

ln119: presented data analysis issues due to progressive technological advancements of neural recordings
ln126: Although they have proposed an
efficient method for spike sorting, it still lacks the speed researchers require
ln130:The larger is the size the slower is the speed and large is the computational time required by spike sorting algorithms. -- rephrase or simply leave out (what, other than the obvious, are you trying to say?)
ln133: They reported, (?)
ln136: These second and third order operations prove the non-linear behaviour of spectral clustering.
ln138: To motivate our analysis,
ln141: The dependency of speed and computational time on data
size in spike-sorting has made it very difficult
ln142: identify the total number of
ln144: breast cancer cell data
ln149: Despite these challenges, ...
ln151: However, limited work has considered enhancing computational
ln153: The proposed algorithm pre-processes data to
ln154: time and to enhance speed and efficiency of a wide range
ln156: by parallel computing approaches to further
ln159: The novelty of the proposed mechanism
ln162:The first step involves subdivision of data into data-subsets of optimal length.
ln164:The second step involves clustering spikes in data-subsets
using conventional spike sorting algorithms.
ln165: The last step involves unification
ln166: clusters are then used to label
ln170:of conventional algorithms but rather performs additional data
ln171: the proposed mechanism very versatile and
ln175: uses a density based
The second step involves clustering
The last step involves unification
ln180: overall time of the spike sorting process.
ln193:The total number N of optimal subdivisions is estimated
ln195, 199: ,where L is the
ln223: of the algorithm depends on the length
ln227: (O L) forms a direct
ln228: and an inverse relationship
the X-axis
the Y-axis
The computational time is the processing time after a movmean
filter (20 datapoints length) filtered the unwanted ripples in the plot and returned smooth curves. (representing computational time (why twice?))
The average value over ten repetitive analyses
robustness of the measure
ln241:'It is observed that, the
variations in data dimensionality does not have any effect on estimating the bounded
region. Whatever is the dimensionality of data, when the ED is calculated, the result is
always a single entry in one-dimensional space. For all EDs The standard deviation
(SD) is calculated using [66] and normal distribution curves are formed based on [67].' --Not even wrong. If you have a multivariate Gaussian distribution, the density distribution as a function of the radius is not Gaussian. The square of the radius (equals the sum of squares of Gaussian

distributed random variables and) follows a Chi-squared distribution (check Wikipedia?) and you can imagine (take the cumulative distribution and rescale the x-axis) what follows for the distribution of the radius itself.
Figure 7: Continues positive trend is observed ???
Errorbars represent...
ln344: To cater for schocastic ??? variations of some of the algorithms
ln335: over 10 repetitons.

Author Response: The manuscript has been thoroughly revised taking into account all the comments by the reviewer.

Thanks

Asim Bhatti
