# Supplementary material for: Efficient neural spike sorting using data subdivision and unification
Source: PLoS One. 2021 Feb 10;16(2):e0245589. doi: 10.1371/journal.pone.0245589 (PMC7875432; doi:10.1371/journal.pone.0245589)
Supplement: Attachment — Submitted filename: Rebuttal Letter_R4.pdf [file pone.0245589.s005.pdf]

PONE-D-19-23638

Efficient Neural Spike Sorting using Data Subdivision and Unification

PLOS ONE

To the Editor,

Gennady Cymbalyuk
Academic Editor, PLOS ONE

We would like to acknowledge and appreciate the efforts and time of the editor and the reviewers for their invaluable comments and suggestions that has allowed us to enhance the quality of our manuscript.

Below are the suggested revisions according to valuable comments from the reviewers.

1) **Lines 237-253: Please talk to a statistician (or someone who knows English and statistics) and reframe (and please put references from peer reviewed publications). This is about making your paper understandable to a reader, and I'm not asking for a layman explanation here (in fact, I feel that you're trying to explain a lot of things you don't need to explain, I'm asking for correctness. The standard deviation of a random variable is a well-known and defined quantity and your equation does not reflect the standard deviation of Euclidean distances.**

   Author Response: The equation has been updated with a square term that was previously missing in the equation. (Please refer to Eq. 7.)

2) **The Euclidean distances are strictly positive numbers, but in Figure 6, you're suggesting that they are Gaussian distributed, and therefore negative values are possible. So I really don't understand what you are doing here.**

   Author Response: Detailed explanation of probability distribution of Euclidean distances is discussed at lines 273 to 318.

3) **And I would also be very interested whether you scale your principal components in some way, to match their variances, or whether the first principal components have larger weights.**

Author Response: The PCA components are not scaled to match their explained variances. The individual variances of PCA components are accumulated and the optimal number of PCA components that gives at least 85% of cumulative explained variance are chosen for the analysis. 10 PCA features are required to get at least 85% cumulative explained variance of the 64 dimensional spikes data. (Lines 353 to 358)

4) **The main issue that I raised in the last revision was that when you're working in a 10 dimensional space, things are a little more complicated. For example, if you have a standard normal distribution in 10 dimensions, then the Euclidean distances (ED) follow a Chi-square distribution with 10 degrees of freedom (see https://en.wikipedia.org/wiki/Chi-square_distribution).**

Author Response: The manuscript is updated with additional information, mathematical expressions, figures and references to address the points raised by the review as well as for the ease of the general readers. (Fig 6, Fig 7 and Lines 234 to 318).

5) **Other comments:**

i. ln. 288 kolmogorov-Smirnov (KS) test --> Kolmogorov-Smirnov (KS) test
ii. ln. 296 Please make clear what you mean by this sentence: 'It is observed that 10 PCA features ensures the cumulative explained variance of over 85% up to 95%, in case of the data sets employed in this study.' Please reframe this sentence.
iii. The matlab function-- Please capitalize MATLAB.

Author Response:
The manuscript has been updated according to reviewer comments.

Thanks

Asim Bhatti
