# Supplementary material for: Efficient neural spike sorting using data subdivision and unification
Source: PLoS One. 2021 Feb 10;16(2):e0245589. doi: 10.1371/journal.pone.0245589 (PMC7875432; doi:10.1371/journal.pone.0245589)
Supplement: Attachment — Submitted filename: Rebuttal Letter_R5.pdf [file pone.0245589.s006.pdf]

PONE-D-19-23638-R5

Efficient Neural Spike Sorting using Data Subdivision and Unification

PLOS ONE

To the Editor,

Gennady Cymbalyuk
Academic Editor, PLOS ONE

We would like to acknowledge and appreciate the efforts and time of the editor and the reviewers for their invaluable comments and suggestions that has allowed us to enhance the quality of our manuscript.

Below are the suggested revisions according to valuable comments from the reviewers.

1) **lines 275-286 and Figure 7: I'd suggest to remove this paragraph and Figure 7 since - I'm not sure how the explanation of a z-score helps in understanding the method. - you are not plotting normal distributions in any of the Figures anymore, you defined the standard deviation in (7), so Equation (9) is not necessary (you're not using any other property of the normal distribution other than its standard deviation, and you cannot assume that the distribution of z scores is normal).**

   Author Response: As per reviewer's suggestion paragraph (lines 275-286) and Figure 7 have been removed.

2) **line 287: A short motivational sentence about what you're planning to do with the z score values would be great, e.g. 'We wanted to determine outliers for each spike cluster. To this aim, we considered two scenarios where the z scores distributions of a given cluster were either consistent with a normal distribution or skewed. There are numerous...'**

   Author Response: The manuscript has been updated and a motivational sentence highlighting the use of Z scores is added at the start of the paragraph. (Please refer to lines 275-281).

3) **if you rather want to keep these lines:**
   a. **line 287: data distribution --> z-score distribution. line 284: Euclidean**
   b. **line 281, 283: 'used to plot', 'is plotted': I don't find these plots anywhere, so please reformulate.**

   Author Response: We have removed the suggested text from the manuscript as per reviewer comments (1 and 2).

Thanks

Asim Bhatti
